# A Study of the Structure, Properties, and Sorption Activity of Oil Sorbents Based on the Secondary Cellulose-Containing Raw Materials of Buckwheat Cereal Production

**DOI:** 10.3390/molecules30112285

**Published:** 2025-05-23

**Authors:** Anton Mostovoy, Natalia Eremeeva, Andrey Shcherbakov, Marina Lopukhova, Sholpan Ussenkulova, Elvira Zhunussova, Amirbek Bekeshev

**Affiliations:** 1 Laboratory of Modern Methods of Research of Functional Materials and Systems, Yuri Gagarin State Technical University of Saratov, Polytechnichskaya St., 77, 410054 Saratov, Russia; 2 Department of Natural and Mathematical Sciences, Yuri Gagarin State Technical University of Saratov, Polytechnichskaya St., 77, 410054 Saratov, Russia; nm.eremeeva-it@yandex.ru; 3 Laboratory of Support and Maintenance of the Educational Process, Yuri Gagarin State Technical University of Saratov, Polytechnichskaya St., 77, 410054 Saratov, Russia; gassmed7@gmail.com; 4 Department of Economics and Humanitarian Sciences, Yuri Gagarin State Technical University of Saratov, Polytechnichskaya St., 77, 410054 Saratov, Russia; mlopuhova@yandex.ru; 5 Department of Chemistry, Chemical Technology and Ecology, K. Kulazhanov Kazakh University of Technology and Business, Kayym Mukhamedkhanov Str., Building 37 A, Astana 010000, Kazakhstan; sholpan_1990@mail.ru (S.U.); tahmina.66@mail.ru (E.Z.); 6 Laboratory of Polymer Composites, K. Zhubanov Aktobe Regional State University, Aliya Moldagulova Avenue 34, Aktobe 030000, Kazakhstan

**Keywords:** buckwheat husk, modification, structure, sorbents, sorption capacity

## Abstract

The possibility of using the secondary cellulose-containing raw material resource of the cereal production of buckwheat, namely, its husk, as sorbents for the collection of oil and oil products is shown. In order to increase the yield of the finished product, develop porosity, and improve the sorption characteristics of the buckwheat husk, methods for its physical and chemical modification are proposed. The effect of the modification modes on the parameters of the porous structure, as well as the sorption capacity of the developed materials for various types of oil products, was studied. The selection of the optimal parameters of the buckwheat husk modification was carried out, ensuring the production of effective unsinkable sorbents based on the buckwheat husk with a reserve buoyancy of more than 20 days and a high sorption capacity of sorbents for oil of up to 6.1 g/g and waste motor oil of up to 4.9 g/g. The use of the buckwheat husk as a sorbent allows not only the elimination of oil pollution on both water and surfaces but also solving the problem of the disposal of agricultural waste.

## 1. Introduction

Modern trends in the development of the chemical industry are characterized, first of all, by an increased emphasis on the operational properties and quality of products, while reducing the cost of their production. This fact explains the growing interest in research on the ways of effectively using various wastes as raw materials.

Products of plant origin, which are accumulated in significant quantities in the form of waste from various industries (the pulp and paper industry and agriculture) are attractive for use in the chemical industry. These materials, due to their high porosity, are of interest as a raw material for obtaining the sorbents used in solving many environmental problems: cleaning gas emissions, waste water, soil, etc. [1,2,3].

In recent years, research on natural organic sorbents has become very popular. In particular, considerable attention is paid to materials based on rice husks [4,5,6,7], bagasse [8,9,10,11], brown coal [12,13], wool [14,15,16,17], walnut shells [18,19,20], various plant fibers [21,22], and straw [23,24]. In addition, studies of oil sorbents based on graphite [25,26] and thermally expanded graphite [27,28] should be noted.

Rice husk is one of the main types of agricultural waste, containing about 70% of lignocellulose material and about 20% of amorphous silicon dioxide—SiO_2_ [29,30]. In this regard, it can be considered one of the renewable and cheap materials for the preparation of carbon-containing composite material. The efficiency of using this particular material as a sorbent of oil from the water surface depends on its adsorption capacity and the rate of oil product sorption. In [31], a porous composite material containing C/SiO_2_ obtained by pyrolysis of rice husk at 480 °C was studied as an adsorbent for removing oil and oil products from the water surface. It was found that the material had a high sorption capacity, good buoyancy, and high hydrophobicity. It was noted that the relationship between the bulk density of the material and the height of the oil product penetrating into it during a certain period of time was inversely proportional, i.e., the higher the density of the carbonized material, the worse the sorption.

It was proposed to use cedar nut shells as a promising raw material for obtaining effective sorbents. In [32], cedar nut shells and hydrolytic lignin were used as a starting material for obtaining microporous activated carbons. It was found that a low heating rate (less than 3 °C/min) promoted the formation of micropores with an average size of about 0.56–0.58 nm at a carbonization temperature of 700 °C, and, at a heating rate of more than 3 °C/min at the same carbonization temperature, the average micropore size was already 0.7–0.78 nm. Steam activation at a temperature of 800 °C led to the formation of a sorption material with a more developed micropore volume (0.3–0.35 cm^3^/g) and a micropore width of about 0.6–0.66 nm, which were capable of separating helium from a mixture of helium and methane. The micropore size varied depending on the degree of burnout.

In recent years, active developments have been carried out to obtain oil sorbents based on forest industry waste for the removal of hydrocarbon spills. The possibility of using birch, pine, and larch bark subjected to explosive autohydrolysis to obtain oil sorbents is given in [33,34,35]. It is noted that in terms of oil capacity and the degree of oil extraction, sorbents are capable of competing with industrial oil sorbents based on peat.

The literature analysis shows that buckwheat production waste has been least studied, and technical proposals for its use are few. At the same time, up to 22% of the total mass of the initial raw material in the processing of grain into cereals is buckwheat husk. Only a small part of it is used as fuel, pillow filling, and the packaging of fragile goods and fruit. Consequently, a huge amount of valuable raw materials of plant origin is renewed annually, which has not found effective use yet [36,37,38].

With such advantages as a low density and attractive specific properties, combined with low cost and renewability, buckwheat husk can be a worthy competitor to both natural and synthetic sorbents.

In this regard, the aim of this work is to develop technology and to select parameters for modifying buckwheat husk to obtain effective oil sorbents and to study their performance characteristics. Methods for the physical and physicochemical modification of buckwheat husks are proposed, including combined heat treatment and impregnation with ammonium tetrafluoroborate, which made it possible to significantly improve its sorption characteristics and increase the yield of the finished product. The effect of the modification modes on the parameters of the porous structure, as well as the sorption capacity of the developed materials for various types of oil products, was studied.

## 2. Results

**Figure 1:**
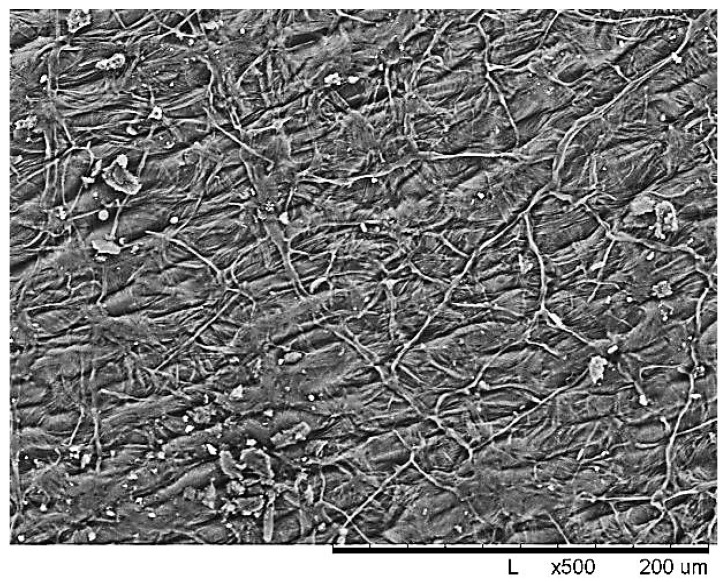
SEM image of surface morphology of raw buckwheat husk.

Buckwheat husk (BH) is a multi-tonnage cellulose-containing renewable agricultural waste obtained directly from a processing plant and cleaned of dust and impurities by dry sieving on a sieve. An analysis of the buckwheat husk structure by scanning electron microscopy Hitachi TM 1000 (Tokyo, Japan) (SEM) shows in Figure 1 that BH is characterized by a rough surface with relief protrusions formed by fibrils oriented in the longitudinal and transverse directions. In general, a volumetric macroporous fibrous structure is formed, which in turn is confirmed by the low value of the bulk density—125 kg/m^3^.

**Figure 2:**
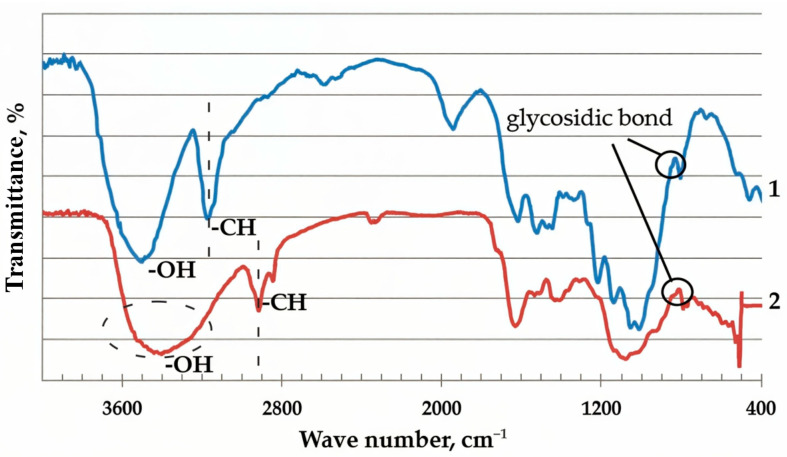
1—FT-IR spectra of cellulose [39,40] and 2—buckwheat husk.

The study of the material by the Fourier IR spectroscopy method showed the presence of an absorption band in the region of 3200–3500 cm^−1^ in the spectra, which indicated the presence of hydrogen-bonded OH– groups in the buckwheat husk (Figure 2). The absorption bands at 2923 cm^−1^ and 2853 cm^−1^ are characteristic of the stretching vibrations of the CH_2_– groups. Stretching vibrations of the glucopyranose ring at 1090 cm^−1^ and the glycosidic –C–O–C– bond at 1060 cm^−1^ and 898 cm^−1^ were also detected [39,40].

Buckwheat husk has high chemical resistance due to its unique composition, in which lignin and cellulose play a key role. Lignin, which forms up to 35% of the buckwheat husk and forms the rigid structure of the plant shells, is practically insoluble in weak acids and retains its integrity even when exposed to aggressive environments [41,42,43]. Cellulose, which forms up to 39% of the husk mass, also makes a significant contribution to its resistance. Although cellulose can be hydrolyzed by strong acids, such as sulfuric acid, it demonstrates high chemical stability at room temperature [42,44,45]. The combination of these two components creates a dense and durable matrix that protects buckwheat husk from destruction under the influence of solvents and acids.

**Figure 3:**
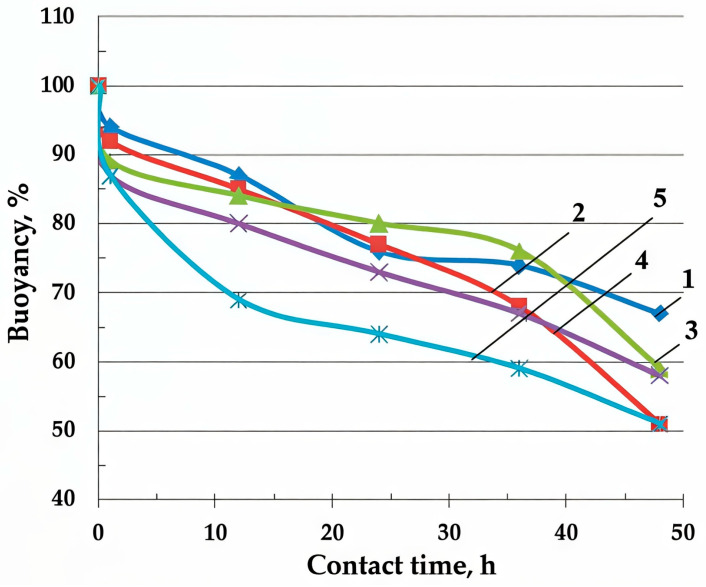
The effects of the layer thickness of the raw buckwheat husk on the time dependence material buoyancy in water (Equation (4)), mm: 1—1–2; 2—3–5; 3—5–7; 4—10; 5—20.

The study of the sorption properties has shown that BH has an insignificant sorption capacity for oil and oil products (0.8–1.0 g/g) and, due to its high hydrophilicity, has low reserve buoyancy. Regardless of the thickness of the BH layer from 1 to 20 mm on the water surface, almost 50% of its sedimentation is observed within 48 h, Figure 3.

To improve the sorption properties of cellulose-containing materials, various modification methods and their combinations are used. An increase in the surface area can be achieved using physical, physicochemical, chemical, and biochemical modification methods. Grinding, increasing porosity, and granulation are the most common and economically feasible methods for increasing the surface area [2,3].

In order to develop porosity, we carried out the physical modification of the material, consisting of heat treatment of the buckwheat husk in the air environment, in the temperature range of 250–700 °C.

During pyrolysis, volatile decomposition products were released from the material. Their qualitative and quantitative assessments were carried out using the gas chromatography method. It was noted that the main products of buckwheat husk pyrolysis were hydrogen, methane, carbon monoxide, and carbon dioxide, Table 1.

**Figure 4:**
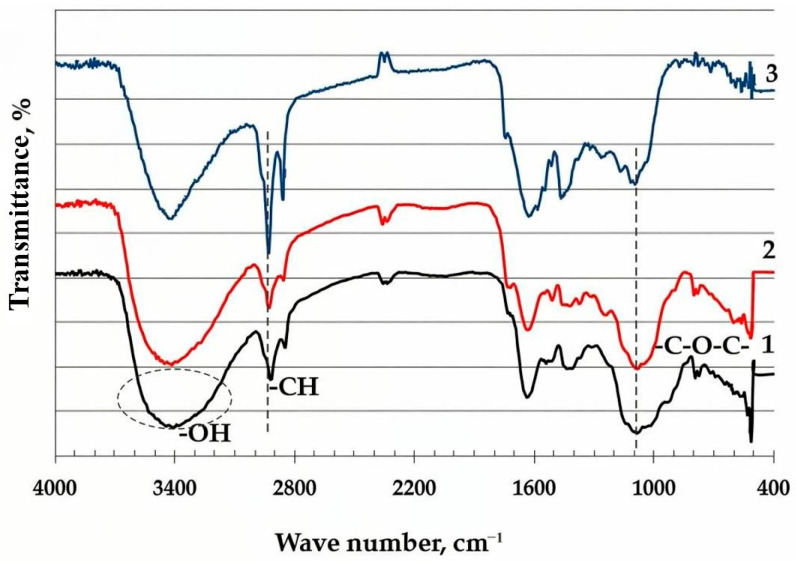
FT-IR spectra of samples: 1—raw buckwheat husk; 2—buckwheat husk heat-treated at 250 °C; 3—buckwheat husk heat-treated at 400 °C.

The thermal modification of BH was systematically investigated using FT-IR spectroscopy to elucidate the chemical transformations occurring at different treatment temperatures: Figure 4. The spectral analysis reveals a clear temperature-dependent evolution of the functional groups, reflecting fundamental changes in the material’s polymeric structure.

At low-temperature treatment (≤250 °C), the FT-IR spectrum exhibits only minor variations compared to the raw material. The preservation of the characteristic absorption bands—including the broad hydroxyl (O–H) stretching vibration at 3400 cm^−1^ and C–H vibrations at 2920 and 2850 cm^−1^—indicates that the primary structural components (cellulose, hemicellulose, and lignin) remain largely intact. However, a slight decrease in the intensity of the O–H band suggests initial dehydration processes.

In contrast, the samples treated above 350 °C demonstrate significant spectral changes indicative of structural reorganization. A sharp decrease in intensity of the O–H band intensity (3400 cm^−1^) was recorded. Simultaneously, the relative enhancement of the C–H stretching vibrations (2920, 2850 cm^−1^) and the prominent aromatic C=C bands (1600–1500 cm^−1^) reflect the formation of new aliphatic intermediates and the progressive aromatization of the residual structure. The observed spectral evolution correlates well with the known thermal degradation mechanisms of lignocellulosic materials, where the depolymerization of hemicellulose (200–300 °C) and cellulose (300–400 °C) precedes the carbonization of lignin-derived components [46,47,48,49].

These spectroscopic findings are particularly significant as they demonstrate that, while low-temperature treatment (≤250 °C) primarily induces physical dehydration, temperatures exceeding 350 °C trigger irreversible chemical transformations, leading to the formation of thermally stable, carbon-enriched structures.

The changes in the structure of the heat-treated (≥350 °C) buckwheat husk are confirmed by the data from the study of the effect of increased temperatures on the husk in the air environment using the thermogravimetric analysis method, Table 2.

The buckwheat husk heat-treated at 250 °C has thermolysis parameters (initial decomposition temperatures and mass losses) similar to the raw husk. Materials treated at 350 °C and above are more heat-resistant. They are characterized by higher initial decomposition temperatures and significantly lower mass losses.

**Figure 5:**
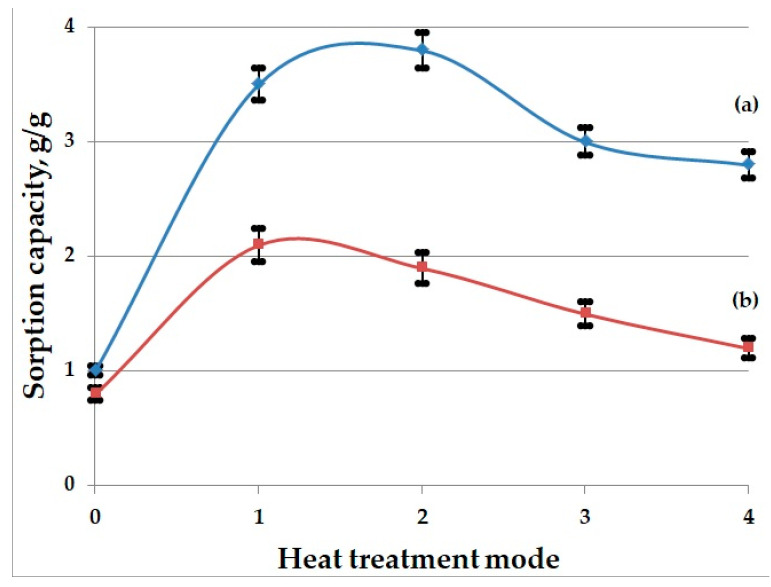
Dependence of sorption capacity ((a)—for oil; (b)—for waste motor oil) on heat treatment temperature mode of buckwheat husk: 0—raw BH; 1—BH heat-treated at 300; 2—BH heat-treated at 350; 3—BH heat-treated at 400; 4—BH heat-treated at 450.

This study of the sorption of oil and waste motor oil shows that the BH heat-treated across the entire temperature range (300–450 °C) sorbs oil products significantly better than the raw husk. The highest sorption rates for oil were found in the samples heat-treated at 350 °C, and for the waste motor oil at 300 °C: Figure 5. As the heat treatment temperature of BH increases, a decrease in sorption capacity is observed.

However, along with sorption, a simultaneous partial release of the oil product from the sorbent volume and the formation of thin films of oil on the surface of the water are visually noted after the sorption completed, i.e., a desorption process was observed. This, in turn, is associated with the difference in the sizes of the molecules of the oil product and the pores of the sorbent, as well as its low specific surface area [50].

**Figure 6:**
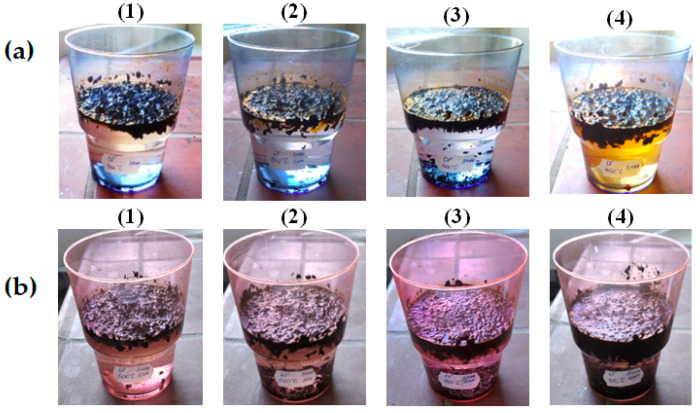
Study of buoyancy of saturated with oil (**a**) and waste motor oil (**b**) BH, heat-treated at temperatures of °C: (1)—300; (2)—350; (3)—400; (4)—450.

It is also noted that there is already a significant sedimentation of the sorbent saturated with the oil product on the bottom of the tank during the first hour of being on the surface. This pattern is especially characteristic of sorbents heat-treated in the range of 350–450 °C, saturated with waste motor oil, the sedimentation of which occurs almost simultaneously with the sorption process, Figure 6.

The results demonstrate that heat treatment significantly affects the sorption capacity of oil products by the BH sorbent. However, this process does not ensure the long-term retention of hydrocarbons within the sorbent’s structure, as partial desorption or leakage may occur over time. Furthermore, the oil-saturated sorbent experiences a loss of buoyancy, which is critical for practical applications in oil spill remediation. This limitation suggests that, while thermal modification enhances the initial uptake, it fails to stabilize the oil–sorbent interaction or maintain the material’s flotation capability. Thus, to improve the performance of sorbents based on BH, additional chemical modification is required.

**Figure 7:**
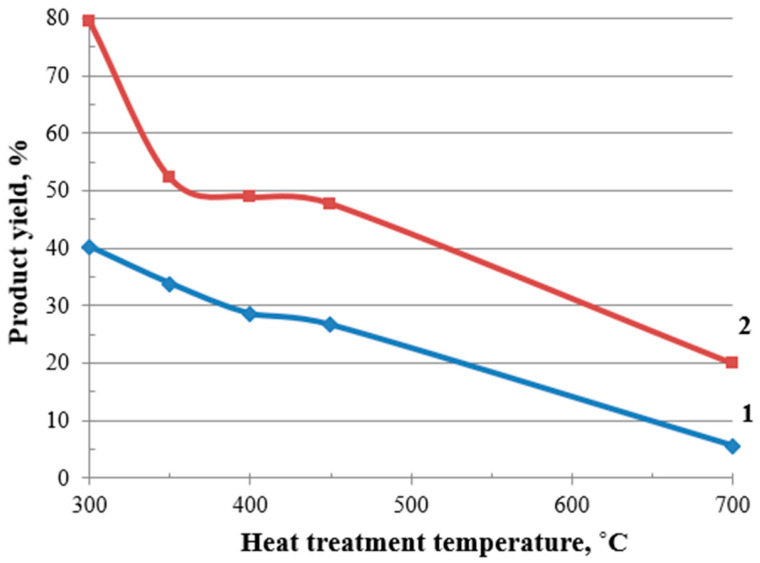
Effect of modification on the material yield: 1—BH; 2—MBH.

Moreover, the experimental results demonstrate a pronounced deterioration in the material yield with an increasing heat treatment temperature. At 300 °C, the process retains a relatively high yield of 40%, indicating moderate structural stability. However, as the temperature rises to 700 °C, the yield plummets to just 6%, suggesting severe degradation of the material’s microstructure, Figure 7.

**Figure 8:**
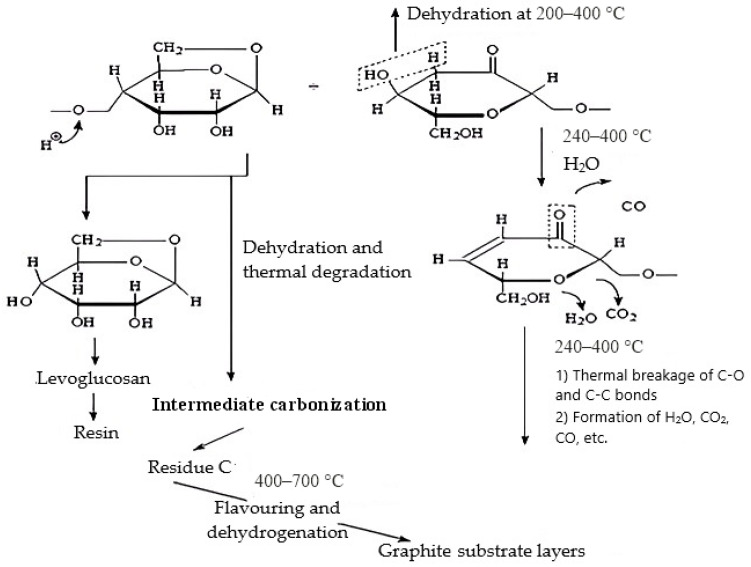
Mechanism of thermal transformations of cellulose, adapted from [46,47,48,49].

During thermal exposure, two types of reactions compete with each other in cellulose-containing materials: condensation, with the formation of carbocyclic structures and water; and destruction, leading to the formation of low-molecular compounds [46,47,48,49]. In this case, the destruction process is accompanied by the formation of levoglucosan; CO, CO_2_, and H_2_O are released during complete destruction and intermediate polyconjugated structures are released during dehydration, Figure 8.

An increase in the yield of carbonized structures is achieved by treating the cellulose-containing material with compounds that act as dehydration catalysts. In this case, the emergence of double bonds and interchain bridges contributes to an increase in the rigidity and thermal stability of the linear macromolecules of the cellulose-containing material and, therefore, to the preservation of their mutually ordered structure [51,52].

In this study, in order to increase the yield of the finished product, the buckwheat husk was modified before the heat treatment. This process consisted of impregnating the material with a 30% aqueous solution of ammonium tetrafluoroborate (TFBA) to obtain modified BH (MBH). The elements contained in TFBA (boron, fluorine, and nitrogen) acted as dehydration catalysts and contributed [53,54] to an increase in the yield of the carbonized structures by 2–4 times: from 40 to 80% at a heat treatment temperature of 300 °C, and from 6 to 20% at a temperature of 700 °C, Figure 7.

The choice of TFBA as a modifying additive was also due to the fact that, based on the thermogravimetric data, TFBA decomposed with a mass loss of 98% in the temperature range of 230–365 °C. Thus, not only an increase in the yield of the finished product by 2–4 times, compared to unmodified BH, was achieved but also a complete absence of the modifier in the composition of the material after its heat treatment. The formation of more heat-resistant structures during heat treatment can be seen in the results of their testing by the TGA method, Table 3. From these data, it is obvious that, when exposed to temperature, the formed structures of MBH were more heat-resistant, since they had a higher thermolysis onset temperature than the raw BH, and this temperature increased with an increase in the heat treatment temperature. They were also characterized by lower mass losses in the entire temperature range studied.

At the same time, it should be noted that in the temperature range of 300–450 °C, structures with approximately the same mass loss indices were formed, i.e., with identical structures. Starting from a temperature of 500 °C, the onset temperatures of thermolysis increased sharply and the mass losses of MBH decreased (Table 3), which indicates the completion of intermediate carbonization and the beginning of the aromatization and dehydrogenation processes, Figure 8.

**Figure 9:**
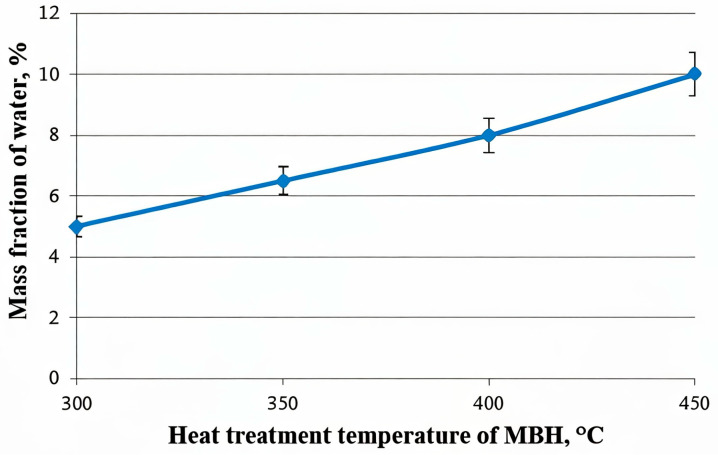
Dependence of moisture absorption on the heat treatment temperature of MBH (heat treatment time—1 min).

The changes in the MBH structure during heat treatment are further supported by the data on water absorption, as illustrated in Figure 9. The graph demonstrates a clear trend: the mass fraction of the absorbed water increases with rising heat treatment temperatures, ranging from 300 °C to 450 °C. This suggests that higher temperatures induce structural modifications in MBH, increasing its porosity.

It is known that the size of an oil molecule ranges from 1 to 4 nm [55]; therefore, a larger pore size of the sorbent reduces the sorption capacity as a result of the predominance of desorption processes over sorption processes in the material, and a smaller one will not allow oil to penetrate into the volume of the sorbent.

In order to select the optimal modification mode, we studied the parameters of the porous structure of the modified buckwheat husk at different heat treatment temperatures. The study of gas adsorption by the modified buckwheat husk can provide valuable information on its specific surface area and porous structure. The method of low-temperature nitrogen adsorption (the BET method) is currently the most widely used for this purpose [56].

Based on the obtained BET data (Table 4), the most developed surface is achieved with the heat treatment of MBH in the temperature range of 350–450 °C. At these heat treatment temperatures, the pore volume increases, but at the same time an increase in the pore radius is noted.

At the same time, a set of model sorbates, methylene blue dye and iodine, which are considered to be “molecular probes” for molecules with sizes of 0.335 nm for iodine [57] and 1.5 nm for methylene blue, were also used to evaluate the porosity of the sorbents. By the value of the iodine sorption activity, the presence of micropores in the sorbent with effective diameters of 0.6–1.5 nm can be seen, and the presence of mesopores with sizes of 1.5–50 nm can be proven by the sorption of methylene blue. Since the molecules sizes are similar, along with the methylene blue dye, the MBH sorption of methyl orange (with a molecule size ~1.8 nm) dye was also studied in order to clarify the results. The activity for methyl orange and methylene blue initially increases with increasing heat treatment temperatures, but in the temperature range of 500–700 °C the values are almost equal, which shows that the formation of the mesoporous structure of the material stops, Table 5.

**Figure 10:**
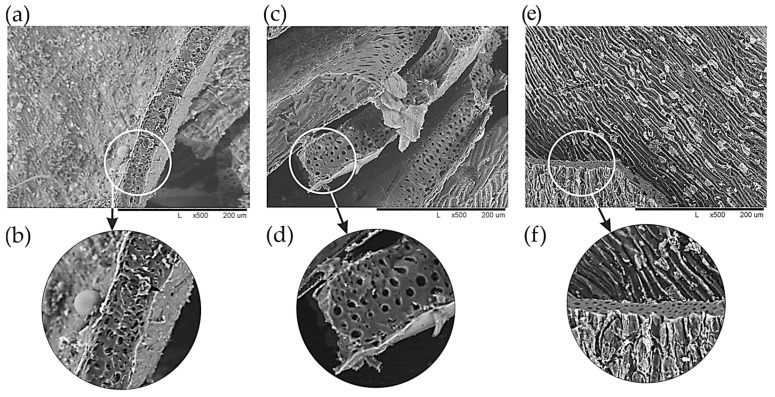
SEM image of the MBH structure heat-treated at temperatures, °C: (**a**,**b**)—350; (**c**,**d**)—500; and (**e**,**f**)—700. (**a**,**c**,**e**)—×500; and (**b**,**d**,**f**)—×1000.

According to the SEM data (Figure 10), an increase in the size of macropores is clearly observed with an increase in the heat treatment temperature to 500 °C. Macropores play the role of transport channels, ensuring the free movement of the adsorbate or reagents (reaction products) inside the porous body. Due to an increase in pore size, the diffusion process occurs more easily, thus increasing the sorption capacity of the material [58,59].

MBH, due to its lignin and cellulose content, retains chemical resistance during heat treatment up to 700 °C, which makes it resistant to acids and solvents [42,60,61]. The heat treatment of MBH at temperatures from 300 °C to 700 °C significantly changes its sorption properties: the sorption capacity for alkali decreases sharply from 12.5 mg-eq/g during heat treatment at 300 °C to 1.3 mg-eq/g at heat treatment temperatures of 400–700 °C, which is probably explained by changes in the chemical composition of the surface. At heat treatments up to 300 °C, a high alkali adsorption capacity (12.5 mg-eq/g) is associated with the acidic groups of lignin and cellulose, but with an increase in the heat treatment temperature up to 350–700 °C, these groups are destroyed, which is also confirmed by FTIR spectroscopy data (Figure 4), reducing the alkali sorption to 1.3 mg-eq/g. At the same time, ammonization using TFBA introduces basic groups, thus increasing acid adsorption from 2.0 mg-eq/g at a heat treatment of 300 °C to 4.6 mg-eq/g at a heat treatment of 700 °C. These changes allow to optimize the use of MBH depending on the target application: MBH heat treated at 300 °C is effective for alkali sorption, and MBH heat treated at 400–700 °C is effective for acids, Table 6.

The volume of the sorption space of sorbents, determined for toluene, is the volume of pores available for the adsorption of toluene molecules, a small organic compound with a kinetic diameter of about 0.6 nm. An increase in this volume with the increasing heat treatment temperature of MBH (Table 7) is associated with an improvement in the textural characteristics of the sorbent, especially with an increase in the volume of micropores (pores with a diameter of less than 2 nm), which are most effective for the adsorption of such small molecules due to their high specific surface area and strong adsorption potential in these limited spaces [62,63].

The main characteristic of a sorbent is its sorption capacity. The study of the sorption capacity was conducted on oil and waste motor oil. The amount of the sorbent in the experiment was determined by the need for the complete removal of the oil product. The thickness of the oil product layer on the water surface varied from 1 to 15 mm.

The sorption capacity of a sorbent depends on the intermolecular penetration of a substance, its ability to retain sorbed substances on its surface, and the degree of synergy of these properties. The priority mechanism of oil sorption is its penetration into pores under the action of capillary forces, while a part of the sorbed substance remains on the surface of the adsorbent under the influence of hydrogen bonds, steric interactions, or other weak interaction forces [50].

**Figure 11:**
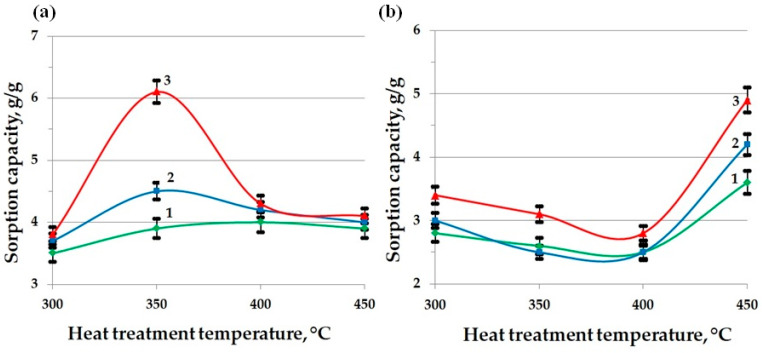
Indicators of the sorption capacity of MBH for oil (**a**) and waste motor oil (**b**), with a layer thickness of oil product, mm: 1—1; 2—5; 3—15.

Figure 11 shows the dependences of the sorption capacity of MBH subjected to a short-term (1 min) thermal modification at temperatures in the range of 300–450 °C, in relation to crude oil (a) and waste motor oil (b). The analysis was performed with varying thicknesses of the oil product layer: 1 mm (curve 1), 5 mm (curve 2), and 15 mm (curve 3). The obtained results demonstrate fundamental differences in the mechanism of the interaction of the sorbent with the studied oil products, caused by changes in the chemical composition and the structural and pore characteristics of the material during the heat treatment.

For crude oil sorption, a pronounced maximum of the sorption capacity (5.9 g/g for a 15 mm layer) is recorded at a heat treatment temperature of 350 °C. This is due to the formation of the most favorable pore space structure in this temperature range, characterized by a high specific surface area (~67 m^2^/g), a large total pore volume (~0.68 cm^3^/g), and an average radius of about 11 nm. In addition, at this stage of thermal modification, functional groups of a polar nature are preserved on the sorbent surface, in particular, hydroxyl (3400 cm^−1^ by FT-IR) and ether (1030–1100 cm^−1^) groups capable of participating in specific adsorption interactions with the heteroatomic components of oil, thus improving the penetration of low-viscosity oil products into the porous matrix.

A further increase in temperature to 400–450 °C leads to a significant restructuring of the pore structure, accompanied by an increase in the average pore radius to 15–18 nm and an almost complete loss of the polar functional groups, which is confirmed by FT-IR spectroscopy (the bands at 3400 and 1030 cm^−1^ practically disappear and the band at 1600 cm^−1^, characteristic of aromatic carbon structures, strengthens). As a result of such structural and chemical changes, the sorption capacity for oil decreases to approximately 4 g/g, since the predominant contribution of specific adsorption interactions disappears and the enlargement of the pore structure becomes less significant for a low-viscosity liquid.

The opposite picture is observed during the sorption of waste motor oil, which has a significantly higher viscosity and contains aggregated additives, soot, and oxidation products. In the low-temperature range (300–350 °C), narrow pores and residual hydrophilicity of the surface create significant kinetic and thermodynamic barriers to the penetration of this product, which is demonstrated by the minimum values of sorption capacity (~2.5–3 g/g). However, in the region of 450 °C, when the pore structure becomes predominantly large mesoporous (with an average radius of about 18 nm) and the surface acquires a pronounced hydrophobic aromatized character, the hydrodynamic resistance to capillary absorption decreases sharply, which leads to a significant increase in the sorption capacity (up to 4.9 g/g for a 15 mm layer). The effect is further enhanced by an increase in the thickness of the oil product layer, ensuring a long-term preservation of the concentration gradient and deeper filling of the pore structure.

Thus, the presented experimental data clearly demonstrate that the optimum sorption capacity of MBH is determined by the balance of geometric (pore size and volume and surface area) and chemical (polarity and surface hydrophobicity) factors. When selecting the temperature mode for the material activation, it is necessary to take into account the viscosity and structural characteristics of the target pollutant: moderately low temperatures (about 350 °C) are preferable for the sorption of low-viscosity crude oil, while higher temperatures (about 450 °C) are optimal for the effective removal of highly viscous waste oils and other complex oil waste.

**Figure 12:**
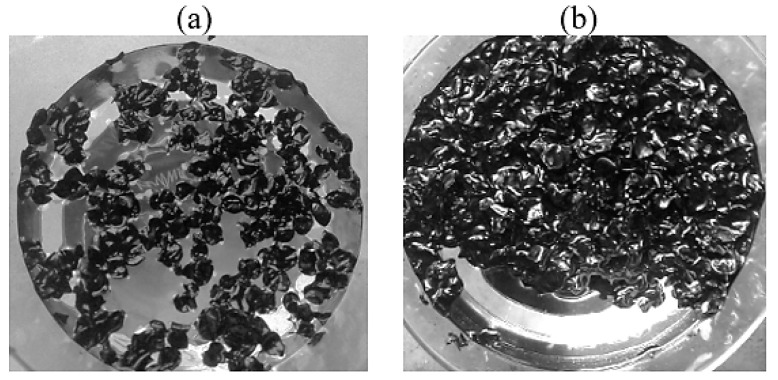
Sorption of oil from the water surface with a layer thickness of (**a**)—1 mm; (**b**)—15 mm.

Based on an increase in the sorption capacity with an increase in the thickness of the oil product layer, it can be seen that the sorption process is not limited to the surface adsorption process, Figure 11. The absorption of oil and oil products by hydrophobic powder materials is not limited to surface sorption. In real conditions, this process dominates in the cleaning of water bodies from monomolecular films of pollutants. When solid oleophilic particles come into contact with a thick film of oil, oil clusters are formed around them, interacting with each other to form a peculiar mesh structure. This results in a significant increase in the viscosity of the suspension as a whole, with the formation of dense conglomerates being observed at high concentrations of powder sorbents in oil [64,65]. In this case, powder hydrophobic materials act as thickening agents, which leads to a decrease in the oil slick area, Figure 12.

The studies of the heat treatment duration of MBH (Table 8) show that, with an increase in the holding time of MBH at a given temperature, the sorption capacity of the material for oil and waste motor oil decreases. This fact indicates the prevalence of desorption processes over sorption processes due to an increase in the pore size in the material.

**Figure 13:**
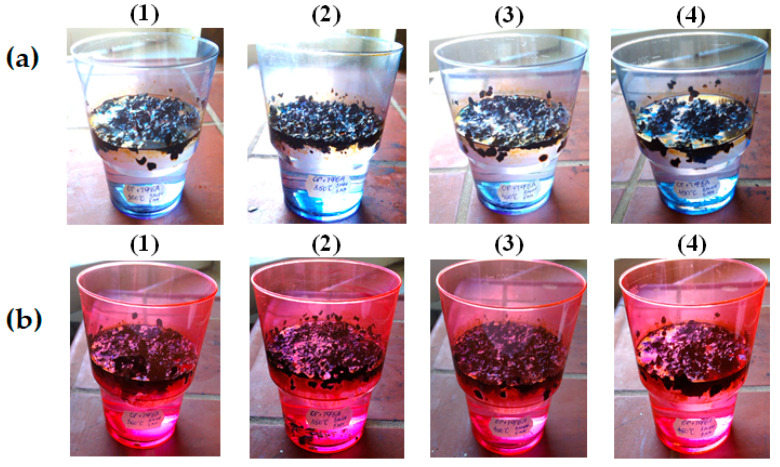
MBH, retaining buoyancy for more than 20 days, saturated with oil (**a**) and used motor oil (**b**), heat-treated at temperatures of °C: (1)—300; (2)—350; (3)—400; (4)—450.

When using materials to remove pollutants from the water surface, such an indicator as buoyancy plays a significant role. The reserve buoyancy should be sufficient until the removal of the waste sorbent is completed. Studies have shown that, after the sorption of oil and waste motor oil, MBH retains buoyancy for more than 20 days, Figure 13.

A comparative analysis of the obtained and existing sorbents (Table 9) shows that buckwheat husk is not inferior or even superior to existing analogues in terms of the main criteria. This fact allows us to say that the obtained sorbent is competitive and can be successfully used to collect oil and waste motor oil from the surface of water.

## 3. Materials and Methods

### 3.1. Materials

Buckwheat hull (BH) is a secondary cellulose-containing renewable raw material resource of buckwheat cereal production. It is represented by particles of 3–4 mm in size and about 1 mm thick. Its bulk density is 125 kg/m^3^.

Light oil with a density of no more than 830 kg/m^3^ at 20° C and waste synthetic motor oil produced by Lukoil (Moscow, Russia) were used as the petroleum products.

Ammonium tetrafluoroborate NH_4_BF_4_ (TFBA), used for the BH modification, is a white crystalline substance, easily soluble in water, with a density of 1.851 g/cm^3^. It was obtained from LLC “REAKHIM” (Moscow, Russia).

### 3.2. Methods

In order to develop porosity, we carried out the physical modification of the material, consisting of heat treatment of the buckwheat husk in the air environment, in the temperature range of 250–700 °C at a temperature rise rate of 10 °C/min. The duration of the process was 1 min, since this was the time during which the integrity of the husk structure was maintained. At a heat treatment temperature of 250 °C, the exposure time was from 60 to 120 min.

In this study, in order to increase the yield of the finished product, the buckwheat husk was modified before the heat treatment. This process consisted of impregnating the material with a 30% aqueous solution of ammonium tetrafluoroborate (TFBA) in a bath module of two, followed by drying at a temperature of 85 ± 5 °C to obtain modified BH (MBH). The choice of a 30% concentration of an aqueous TFBA solution for the modification ensured the TFBA content in the cellulose was the amount of 35 wt.%, which was due to the need to add nitrogen into the cellulose composition as a combustion inhibitor to the amount of at least 4.5%, and its synergetic—boron—to at least 7% [53,54]. An increase in the amount of 35 parts by mass was possible with a bath module equal to two, leading to the complete absorption of the impregnating bath, which, in turn, prevented the formation of wastewater.

The study of the surface morphology and structure of the carbon materials was carried out using the Hitachi TM 1000 scanning electron microscope (Tokyo, Japan).

The study of the surface structure of the material was carried out using the Axio Imager.A2m optical microscope with an objective Achroplan from Carl Zeiss AG (Carl Zeiss Microscopy LLC, New York, NY, USA).

FT-IR spectroscopy of the samples was carried out using the IRTracer-100 device from Shimadzu (Tokyo, Japan).

A pyrolysis unit was used to obtain the pyrolysis gases, which were subsequently analyzed by gas chromatography. The sample was ground and weighed, then loaded into the container, which was subsequently placed in the reactor. Heating was carried out without air access, in the temperature range of 20–700 °C, until the release of the reaction products ceased. Hydrogen sulfide and hydrocarbons (C_2_-C_5_) were determined using a column filled with Paropak Q (Waters Corporation, Milford, MA, USA); hydrogen, methane, carbon monoxide (II), and carbon monoxide (IV) were determined using a column filled with activated carbon with 25% of iodine.

A thermogravimetric analysis of the samples was carried out using a MOM Q-1500D derivatograph (Budapest, Hungary). The analysis was conducted in air, and the relative error did not exceed 1%.

The study of the porous structure of the samples was carried out using the Nova 1200e apparatus (Nova Biomedical, Waltham, MA, USA) by the low-temperature nitrogen adsorption method.

The mass fraction of water in the sample was determined in accordance with the standard [73].

To determine the adsorption activity of the sorbents using methylene blue and methyl orange indicators, a sample (0.1 g) of pre-dried sorption material was placed in a 50 cm^3^ conical flask, then 25 cm^3^ of the indicator solution was added, and the flask was closed with a stopper and shaken using an apparatus for shaking liquid in vessels for 20 min. After shaking, the suspension was placed in centrifugation tubes and centrifuged for 15 min, and 5 cm^3^ of the clarified solution was carefully pipetted and its optical density was determined using a photoelectrocolorimeter with a blue light filter with a wavelength of 400 nm in cuvettes with a light-absorbing layer thickness of 10 mm. Distilled water was used as a control solution. If the optical density of the clarified solution exceeded 0.8 optical units, then 5 cm^3^ of this solution was placed into a measuring flask with a capacity of 25 or 50 cm^3^, depending on its optical density. The solution in the flask is diluted with distilled water to the mark. The optical density of the solution after dilution should be from 0.1 to 0.8 optical units. The dilution factor was equal to 5 or 10.

The residual mass concentration of the indicator in the clarified solution was determined from the obtained optical density value using the calibration graph. Comparison solutions were prepared to build the calibration graph.

The adsorption activity for the indicator (X) was calculated using the following formula:

$$X=\frac{(C_{1}-C_{2}\cdot K)\cdot 0.025}{m}$$

where C_1_ is the mass concentration of the initial indicator solution, mg/dm^3^; C_2_ is the mass concentration of the solution after contact with the sorbent, mg/dm^3^; K is the dilution factor of the solution taken for analysis after contact with the sorbent; m is the mass of the sorbent sample, g; and 0.025 is the volume of the indicator solution, dm^3^.

The result of the analysis was taken as the arithmetic mean of the results of two parallel determinations, the absolute discrepancy between which did not exceed the permissible discrepancy, equal to 10 mg per 1 g of product.

To determine the adsorption activity of the sorbents for iodine, a sample (1 g) of pre-dried sorption material was placed in a 250 cm^3^ conical flask, 100 cm^3^ of iodine solution in potassium iodide was added, and the flask was closed with a stopper and shaken continuously for 15 min at an intensity of at least 100–125 vibrations per minute. Then, the solution was allowed to settle and 10 cm^3^ of the solution was taken from the flask with a pipette, placed in a 50 cm^3^ conical flask, and titrated with a sodium thiosulfate solution. At the end of the titration, 1 cm^3^ of starch solution was added and titrated until the blue color disappeared. At the same time, the initial iodine content in the solution was determined. For this purpose, 10 cm^3^ of iodine solution in potassium iodide was taken and titrated with a sodium thiosulfate solution, adding a starch solution at the end of the titration.

The adsorption activity of the material for iodine (X) in percent was calculated using the following formula:

$$X=\frac{(V_{1}-V_{2})\cdot 0.0127\cdot 100\cdot 100}{10\cdot m}$$

where V_1_ is the volume of sodium thiosulfate solution (0.1 N) used for the titration of 10 cm^3^ of iodine solution in potassium iodide, cm^3^; V_2_ is the volume of sodium thiosulfate solution (0.1 N) used for the titration of 10 cm^3^ of iodine solution in potassium iodide after treatment with the sorbent, cm^3^; 0.0127 is the mass of iodine corresponding to 1 cm^3^ of sodium thiosulfate solution (0.1 N), g; 100 is the volume of iodine solution in potassium iodide, cm^3^; and m is the mass of the sorbent sample, g.

The result of the analysis was taken as the arithmetic mean of the results of two parallel determinations, the absolute discrepancy between which did not exceed the permissible discrepancy equal to 3%.

The sorption capacity of BH for alkali (acid) was determined as follows: 0.5 g of the material was placed in the flask, 50 cm^3^ of a 0.1 N solution of caustic soda (a 0.1 N solution of hydrochloric acid) was added, and the mixture was shaken for 60 min. Then, 10 cm^3^ of the solution were placed in the conical flask and titrated with a 0.1 N solution of hydrochloric acid (a 0.1 N solution of caustic soda) in the presence of a phenolphthalein indicator. At the same time, a blank experiment was carried out, for which 10 cm^3^ of a 0.1 N solution of alkali (hydrochloric acid) was titrated with a 0.1 N solution of hydrochloric acid (caustic soda) in the presence of a phenolphthalein indicator. The sorption capacity, mg-eq/g, was calculated using the following formulas: (1)—for acid; (2)—for alkali.

(1) $$A=\frac{(V_{1}-V_{2})\cdot 1.33}{m\cdot (1-W)}$$

(2) $$A_{1}=\frac{(V_{1}-V_{2})\cdot 1.15}{m\cdot (1-W)}$$

where V_1_—the volume of 0.1 N of hydrochloric acid or alkali solution used for the titration; V_2_—the volume of 0.1 N of hydrochloric acid or alkali solution used for the titration of 10 cm^3^ of caustic soda or hydrochloric acid solution after treatment with these BH solutions, cm^3^; 1.33 and 1.15—the correction factors for acid and alkali, respectively; W—the mass fraction of water in the sample, %; and m—the sample mass, g [74].

The volume of the sorption space for toluene vapor was determined using the following method. An absolutely dry sample, weighed on the analytical balance to an accuracy of 0.0002 g, was placed in the weighing bottle, previously brought to a constant mass and weighed (to an accuracy of 0.0002 g). The weighing bottle with the sample, with the lid open, was placed in the desiccator filled with toluene vapor and was kept there for 5 h. After the time had elapsed, the weighing bottle was tightly closed with the lid, removed, and weighed on the analytical balance to an accuracy of 0.0002 g. The volume of the sorption space was calculated using the following formula:

(3) $$V=\frac{\left(m_{2}-m_{1}\right)}{\rho \cdot (m_{1}-m_{0})}$$

where V is the volume of the sorption space, cm^3^/g; m_0_ is the mass of the empty weighing bottle; m_1_ is the mass of the absolutely dry sample; m_2_ is the mass of the sample after the sorption of toluene vapor, g; and ρ is the density of toluene, g/cm^3^.

To determine the buoyancy of the sorbent, sorbent samples with a mass m_1_ from 0.25 to 3.20 g were placed in 250 mL beakers half-filled with water. The thickness of the sorbent layer in the beakers was 1–2; 3–5; 5–7; 10, and 20 mm. After the time specified for each series (12, 24, 36, and 48 h) had passed, the remaining sorbent afloat was removed from the surface of the water and dried in the drying cabinet to a constant mass m_2_ at a temperature of 105 ± 1 °C. The amount of sunken sorbent m^3^ was determined by the difference in masses.

The buoyancy X was determined by the following formula:

(4) $$X=\left(\frac{m_{1}-m_{2}}{m_{1}}\right)\cdot 100\%$$

The main property of oil sorbents that determines their efficiency is their sorption capacity, but, in practice, it is necessary to take into account the operating conditions that can significantly affect its value. One of these conditions is an aqueous environment [67]. The efficiency of the sorbents was estimated using the gravimetric method [67]. A sample of the oil product (m1) was applied to the water surface in the beaker filled with distilled water with an adjustable layer thickness. Then, the sorbent was applied to the surface of the oil product to an amount (m0). The system was left at room or laboratory temperature (25 °C) for 15 min to ensure cleaning without stirring. At the end of the 15 min period, a sample of the saturated sorbent was removed using a stainless steel sieve and left to drain for 8 min. After that, the saturated sorbent was weighed (m2).

The sorption capacity X, g/g, was calculated by the following formula:

(5) $$X=\frac{m_{2}-m_{0}}{m_{0}}$$

## 4. Conclusions

As a result of the conducted research, the parameters for obtaining the sorbents for oil and oil products based on buckwheat husk were selected. The chemical composition, structure, physical, and physicochemical properties of the raw and modified buckwheat husk were studied. The possibility of the targeted regulation of the structure, properties, and ability to carbonize buckwheat husk with the complex use of physical and physicochemical modification methods has been proven, which allows the formation of a structure with a given porosity by changing the modification conditions (the heat treatment temperature, duration of thermal exposure, and use of structuring modifiers).

The choice of ammonium tetrafluoroborate as a modifier of the cellulose-containing materials, which helps increase the yield of the main product during heat treatment, has been substantiated. The optimal parameters for modifying buckwheat husk were selected, ensuring the production of effective unsinkable sorbents based on buckwheat husk with a reserve buoyancy of more than 20 days and a high sorption capacity of sorbents for oil of up to 6.1 g/g and waste motor oil of up to 4.9 g/g.

The use of buckwheat husk as a sorbent makes it possible not only to eliminate oil pollution on both water and soil surfaces but also to solve the problem of agricultural waste disposal.

## Figures and Tables

**Table 1:** Volatile decomposition products of buckwheat husk.

Temperature, °C	Components	Volume Fraction, % vol.	Mass fraction, % Mass.
250–315	H_2_	0.6	0.1
	CO	13.6	9.6
	CH_4_	0.3	0.1
	CO_2_	72.4	80.4
315–438	H_2_	0.1	0.1
	CO	22.6	16.1
	CH_4_	0.4	0.2
	CO_2_	69.5	77.9
438–560	H_2_	0.4	0.1
	CO	25.4	18.8
	CH_4_	4.3	1.8
	CO_2_	62.5	72.8
560–700	H_2_	5.2	0.3
	CO	18.1	16.8
	CH_4_	28.4	15.1
	CO_2_	40.3	58.9

**Table 2:** The results of TGA of raw and heat-treated buckwheat husk.

Sample	T_i_–T_f_, °C	m_i_–m_f_, %	Mass Loss, %, at Temperatures				
			200 °C	300 °C	400 °C	500 °C	600 °C
raw BH	200–410	10–55	13	43	54	64	70
BH heat-treated at 250 °C for 90 min	200–325	7–48	7	45	57	64	69
BH heat-treated at 250 °C for 120 min	215–365	6–45	6	37	52	61	66
BH heat-treated at 350 °C for 1 min	370–900	7–93	5	6	8	33	50
BH heat-treated at 700 °C for 1 min	675–930	5–17	5	5	5	18	25

Note: T_i_ and T_f_ are the initial and final temperatures of the main stage of thermolysis and m_i_ and m_f_ are the mass losses corresponding to them.

**Table 3:** The results of the TGA of MBH.

Sample	T_i_–T_f_, °C	m_i_–m_f_, %	Mass Loss, %, at Temperatures				
			200 °C	300 °C	400 °C	500 °C	600 °C
Raw BH	200–410	13–55	13	43	54	64	70
MBH heat-treated at 300 for 1 min	345–750	11–91	7	8	19	41	66
MBH heat-treated at 350 for 1 min	350–790	10–91	7	8	14	35	60
MBH heat-treated at 350 for 10 min	400–790	13–88	7	9	14	31	56
MBH heat-treated at 450 for 1 min	390–860	16–84	14	15	18	30	52
MBH heat-treated at 450 for 10 min	420–790	17–86	11	12	16	28	52
MBH heat-treated at 500 for 1 min	540–925	16–59	10	12	13	15	21
MBH heat-treated at 600 for 1 min	685–930	6–37	1	2	2	3	4

Note: T_i_ and T_f_ are the initial and final temperatures of the main stage of thermolysis and m_i_ and m_f_ are the mass losses corresponding to them.

**Table 4:** Effect of heat treatment temperature on structural parameters of MBH.

Sample	Surface Area, m^2^/g	Pore Volume, cm^3^/g	Average Pore Radius, nm
Raw BH	0.27	0.001	2.7
MBH heat-treated at 250 °C	6.40	0.021	2.9
MBH heat-treated at 350 °C	67.00	0.680	10.8
MBH heat-treated at 400 °C	80.20	0.540	14.9
MBH heat-treated at 450 °C	61.01	0.360	18.3
MBH heat-treated at 700 °C	6.02	0.210	5.9

**Table 5:** Characteristics of adsorption properties of MBH.

Temperature of Heat Treatment of MBH, °C	Adsorption Activity for Iodine, %	Adsorption Capacity for Methylene Blue, mg/g	Adsorption Capacity for Methyl Orange, mg/g
300	14	50	66
350	21	55	72
450	21	108	112
500	25	129	121
600	32	132	120
700	37	130	119

**Table 6:** Sorption capacity of MBH for electrolytes.

Heat Treatment Temperature, °C	Sorption Capacity for Alkali, mg-eq/g	Sorption Capacity for Acid, mg-eq/g
300	12.5	2.0
350	2.5	2.0
400	1.3	2.3
450	1.3	2.9
700	1.3	4.6

**Table 7:** Volume of sorption space of MBH for toluene.

Heat Treatment Temperature, °C	Heat Treatment Time, min	Volume of Sorption Space, cm^3^/g
250	90	0.00580
300	1	0.00476
350	1	0.01047
450	1	0.02350
700	1	0.08323

**Table 8:** The influence of heat treatment duration on the sorption capacity of MBH.

Heat Treatment Temperature, °C	Duration of Heat Treatment, min				
	1	3	5	7	10
Sorption capacity for oil, g/g					
350	4.5	3.4	3.1	3.1	2.9
Sorption capacity for waste motor oil, g/g					
450	4.2	3.6	2.9	2.7	2.2

Note: the layer thickness of the oil product was 5 mm.

**Table 9:** Comparison of the sorbent obtained with analogues.

Sorbent	Surface Area, m^2^/g	Pore Volume, cm^3^/g	Adsorption Capacity for Oil, g/g
Sorbent based on buckwheat husk	67	0.68	6.1
Analogues			
Sorbent based on wheat straw [66]	-	-	6.9
Sorbent based on flax fibers [56]	75.8	0.433	17.4
Sorbent based on plantain leaves [67]	1008.5	0.95	12.8
Modified cornstalk biochar [68]	1.8	0.00225	8.7
Sorbent based on lignocellulosic biomass [69]	-	-	18.0
Fe_3_O_4_ composite nanoparticles [70]	935.9	0.25	8.0
Magnetic titania nanotubes [71]	-	-	1.8
Sorbent based on groundnut husks [72]	-	-	1.1
Sorbent based on banana trunk fiber [72]	-	-	2.1
Sorbent based on rice husks (carbonized) [72]	-	-	3.7

## Data Availability

The data are contained within this article.
